# Evaluation of Osteopathic Principles in Cadaveric Specimens Using Radiological Assessment

**DOI:** 10.7759/cureus.32120

**Published:** 2022-12-01

**Authors:** Imran Siddiqi, Alice Wang, Max Marino, Ira Bowen, Dan E Miulli

**Affiliations:** 1 Department of Neurological Surgery, Riverside University Health System Medical Center, Moreno Valley, USA; 2 Department of Neurological Surgery, Western University of Health Sciences, Pomona, USA; 3 Department of Neurosurgery, Arrowhead Regional Medical Center, Colton, USA

**Keywords:** neuropathophysiology, neurophysiology, neuro, osteopathic manipulative treatment, osteopathic manipulative therapy, somatic dysfunction, fryette principle, osteopathic

## Abstract

Background: Osteopathic manipulative treatment (OMT) of the spine requires the physician to first be able to diagnose with palpation of the spinous processes, transverse processes, and facets, test for movement of the anatomy, and evaluate changes in tissue texture at each level. Physicians should then apply these changes to Fryette’s Principles to effectively understand the corresponding somatic dysfunction and provide treatment. Continuing education in osteopathic principles and practices is important throughout an osteopathic physician’s training.

Aim: Diagnosis and treatment require an understanding of the complex neuroanatomy and physiology of patients. We sought to evaluate the diagnostic capabilities of osteopathic physicians. This was done by evaluating the accuracy of diagnosis of somatic dysfunction on a cadaver specimen and verifying via fluoroscopy and blunt dissection.

Materials & Methods: Fresh refrigerated cadavers were palpated for lesions in the thoracic spine by residents and attendings, and diagnoses of somatic dysfunction were made. Anterior-posterior X-rays were taken with a C-arm. These levels were then exposed by blunt dissection, and somatic dysfunctions were visualized and recorded. Comparative analyses were conducted to evaluate the accuracy of diagnosis.

Results: The accuracy of diagnoses was correct in those who had OMT skills reassessed throughout training and continuing medical education. Osteopathic physicians who routinely kept up with their training were better able to make diagnoses of somatic dysfunction.

Conclusion: Continuing osteopathic medical education with an emphasis on the maintenance of palpatory skills is important. Those physicians with the greatest accuracy of somatic dysfunction diagnosis were physicians who routinely underwent reassessment and continuing medical education of osteopathic skills.

## Introduction

Osteopathic manipulative treatment (OMT) of the spine requires the physician to palpate the spinous processes, transverse processes, facets, and soft tissue and to test for specific movement and changes in tissue texture at each level. Once a diagnosis is made (for example, side-bent left, rotated right), the physician used an understanding of anatomy and physiology (that this needs to be corrected to be rotated left and side-bent right to be put back to proper neutral position), so that the body can be in the proper position and hence function according to normal physiology. While these techniques provide great relief to many patients, anatomical and cadaver studies to assess and correlate the physician’s palpatory skills have been limited. Throughout the history of osteopathic medicine, periodic review of previous principles as well as synthesis within the framework of currently accepted biological understanding has occurred [1]. We sought to continue this tradition with an assessment of osteopathic principles in cadaveric studies that were corroborated by direct anatomical and radiographic evidence.

Somatic dysfunction is an osteopathic concept used to designate the impaired or altered function of the related components of the somatic system [2]. Previous literature has cited unclear pathophysiology and poor reliability of detection [3]. To assess the ability of physicians to palpate and diagnose spinal somatic dysfunction, a group of physicians palpated four cadaver spines and recorded their diagnoses. Palpation involved the assessment of the spinous processes as well as the rotation of individual vertebrae by assessing posteriorly rotated transverse processes and their associated ligamentous and muscular attachments. Cadavers were in the prone position. Identification of somatic dysfunction via evaluation of the paraspinal musculature has been shown to be the primary method for the identification of segmental somatic dysfunction [4].

Following palpation by the physicians, this diagnosis was then compared with radiographic findings as well as visual findings following muscle removal and exposure of the bony ligamentous elements. An assessment was then made between the correlation of the anatomical and radiographic findings and the palpatory diagnosis. Testing of motion at each level was also performed in order that Fryette’s Principles could be observed when the spine was flexed, extended, or neutral.

## Materials and methods

This study was approved by the Arrowhead Regional Medical Center Institutional Review Board (protocol #22-49). Resident physicians (PGY1, PGY3, PGY7) and one attending physician trained in OMT evaluated four cadavers over a two-day course. Residents were part of Riverside University Health System’s Neurological Surgery program. Osteopathic manipulative medicine (OMM) and OMT were a regular part of the didactic sessions of this program, and resident physicians’ skills were routinely assessed and encouraged throughout their training. Osteopathic principles and practice (OPP), OMM, and OMT were utilized by resident physicians and attendings in the clinical care of patients.

**Figure 1:**
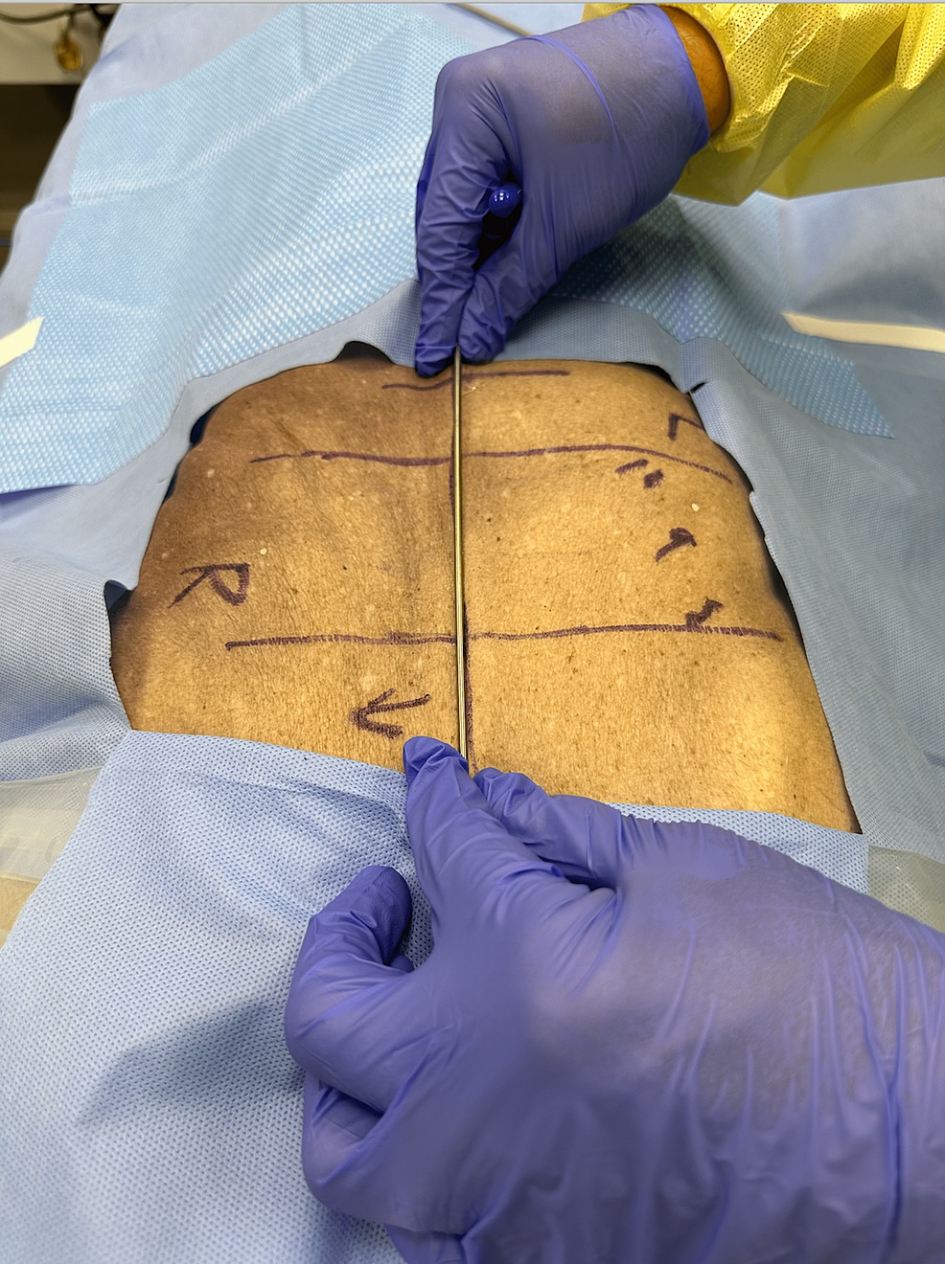
Metal rod utilized to mark the midline of cadaver for radiographic evaluation of somatic dysfunction.

Fresh refrigerated cadavers were palpated for lesions at the T8, T9, and T10 levels by residents and attendings. On the first day, physicians (three residents, one attending) participated and evaluated two cooled cadavers. Each resident first palpated the cadaver at thoracic levels T8-T10 and wrote down a somatic dysfunction diagnosis on a pre-made anonymous form. Anterior-posterior X-rays were taken with a C-arm utilizing a metal rod to mark the midline (Figure 1). Next, the residents localized the thoracic levels using fluoroscopy and sharply exposed levels T8-T10. There was no blunt dissection. There was no pressure put upon the anatomical structures except for the scalpel along the muscle attachment to the structures. Sharp dissection preserved all ligaments, tendons, and facet joints. Any visible abnormalities, including ligamentous hypertrophy and asymmetry indicating side bending or rotation, were identified, preserved, and recorded. Additional anterior-posterior X-rays were taken with the midline marker after dissection to determine whether there was any change in the previously identified somatic dysfunction. Anatomical measurements of the inter-facet distances were recorded. Photos using retractors placed under direct vision to not change alignment were taken to record any asymmetry seen after exposure. On the second day, the same process was repeated on two more cadavers, again using palpation to make diagnoses of somatic dysfunction by the same number of residents and attending physicians.

## Results

Cadaver 1

**Figure 2:**
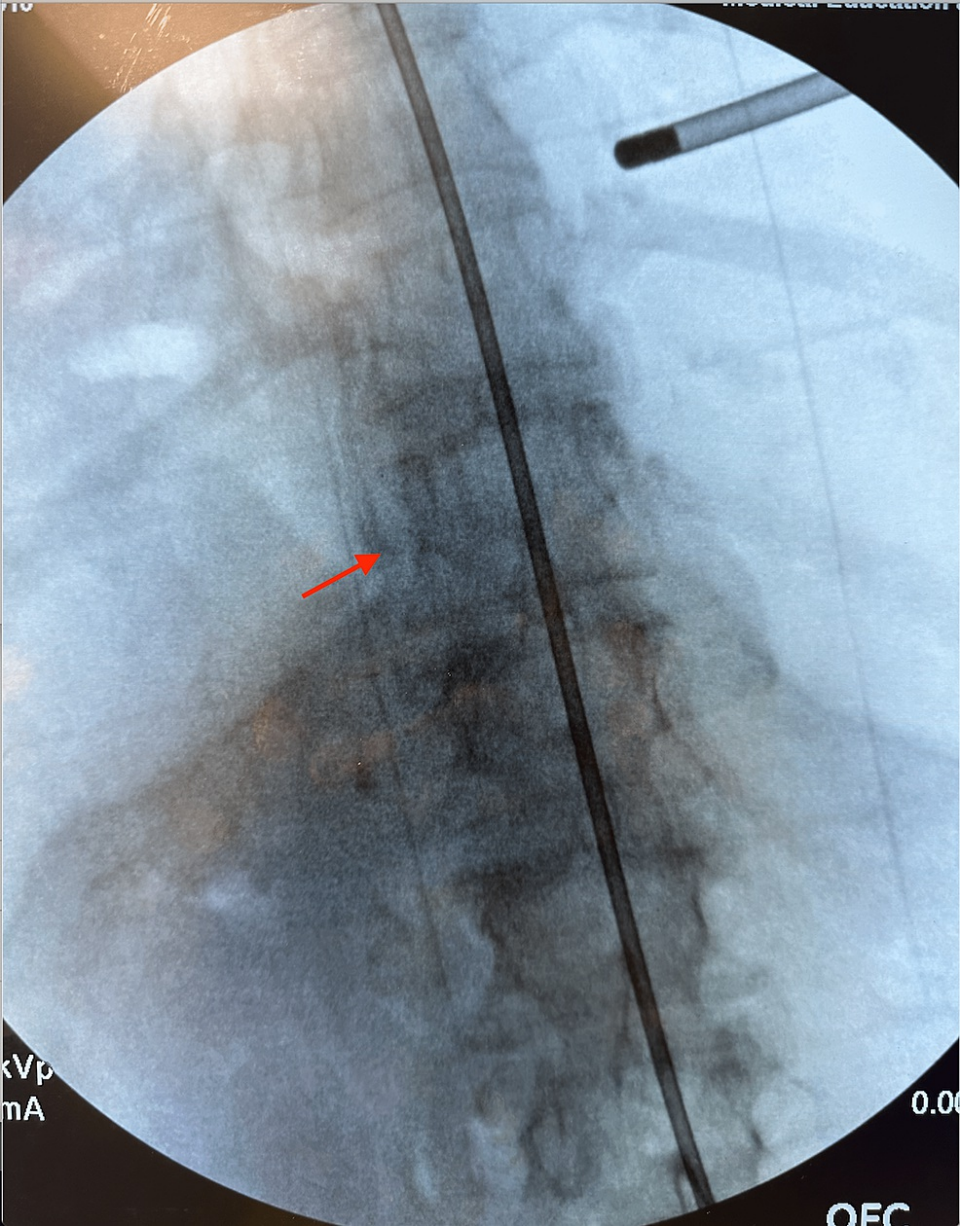
Cadaver 1, demonstrating vertebral bodies rotated right, with slight side bending to the left. Red arrow signifies the patient's left side.

On fluoroscopy, the vertebral bodies appear to be rotated right, with a slight side bending to the left (Figure 2). When the side-bending diagnosis using inter-facet distance was compared to the diagnosis obtained by palpation, 100% were consistent at T8-T9 and 100% were consistent at T9-T10 (Table 1). However, the three most experienced practitioners’ diagnoses were consistent with the inter-facet distance 100% of the time. There was no change in the positioning of the vertebral bodies on post-exposure fluoroscopy. Towel clamps were used to move the vertebral bodies from a flexed position to a neutral position, which corrected the rotation and side bending, thus demonstrating in human cadavers the utility of OMT and all Fryette’s Principles. When the vertebral segments were in a flexed position, they were rotated right side and side-bent right. When the vertebral segments were returned to a neutral position, the rotation and side bending resolved. This again supports Fryette’s second and third principles of osteopathy [5].

**Table 1:** Evaluation of somatic dysfunction of cadaveric specimen levels, inter-facet distances, and accuracy of diagnoses as confirmed via radiographic assessment.

Specimen number	Levels	Diagnosis based on inter-facet distance (side bending)	Inter-facet distance: left (mm)	Inter-facet distance: right (mm)	% Palpatory diagnosis correlating with inter-facet distance
Cadaver 1	T8-T9	Left	11	17	100%
	T9-T10	Right	14	12	100%
Cadaver 2	T8-T9	Right	14	9	100%
	T9-T10	Left	11	18	100%
Cadaver 3	T8-T9	Neutral	11	11	100%
	T9-T10	Right	13	6	100%
Cadaver 4	T8-T9	Left	9	11	100%
	T9-T10	Right	11	10	75%

Cadaver 2

On fluoroscopy, the vertebral bodies appear to be rotated right, side-bent right at T8/T9, and rotated left, side-bent left at T9/T10. This is consistent with 100% of diagnoses at T8-T9 and 100% of diagnoses at T9-T10. The three most experienced practitioners’ diagnoses were consistent with the inter-facet distance 100% of the time. There was no change in the positioning of the vertebral bodies on post-exposure fluoroscopy. Towel clamps were again used to move the vertebral body segments from a flexed position to a neutral position, which corrected the rotation and side bending into a neutral position again supporting Fryette’s second and third principles of osteopathy that side bending and rotation are to the same side in a flexed or extended position and motion in one plane will change the motion in all the others. 

Cadaver 3

On fluoroscopy, the vertebral bodies appear to be rotated right, side-bent left at T8/T9, and rotated left, side-bent left at T9/T10. Although side bending was appreciated on palpation by all practitioners, the inter-facet distance was equal at T8/T9 between left and right. At T9/T10, the inter-facet distance difference was consistent with right side bending. This was 100% consistent with the palpatory findings. Further, there was no change in the positioning of the vertebral bodies on post-exposure fluoroscopy, which supports the initial diagnosis.

Cadaver 4

On fluoroscopy, the vertebral bodies appear to be rotated right, side-bent left at T8/T9, and rotated left, side-bent left at T9/T10. The palpatory diagnoses were 100% correct at T8/T9 and 75% correct at T9/T10, with one junior resident unable to appreciate a non-neutral somatic dysfunction. There was no change in the positioning of the vertebral bodies on post-exposure fluoroscopy, which supports the diagnoses made. The preceding evaluations are tabulated in Table 1. In all cadaveric specimens, final somatic dysfunctions as assessed by each resident can be found in Table 2. 

**Table 2:** Final somatic dysfunctions of thoracic levels T8-T10 in cadaveric specimens as assessed by the residents and attending for the evaluation of cadaveric somatic dysfunction. F: flexed; E: extension; N: neutral; S: side bend; R: rotation; PGY: postgraduate year Residents 1-4 correspond to PGY1, PGY3, PGY7, and attending, respectively

	Resident	T8	T9	T10
Cadaver 1	1	F R_L_ S_L_	F R_R_ S_R_	F R_R_ S_R_
	2	F R_L_ S_L_	F R_R_ S_R_	F R_R_ S_R_
	3	F R_L_ S_L_	F R_R_ S_R_	F R_R_ S_R_
	4	F R_L_ S_L_	F R_R_ S_R_	F R_R_ S_R_
Cadaver 2	1	F R_R_ S_R_	F R_L_ S_L_	N R_L_ S_R_
	2	F R_R_ S_R_	F R_L_ S_L_	N R_L_ S_R_
	3	F R_R_ S_R_	F R_L_ S_L_	N R_L_ S_R_
	4	F R_R_ S_R_	F R_L_ S_L_	N R_L_ S_R_
Cadaver 3	1	N R_R_ S_L_	E R_R_ S_L_	E R_R_ S_L_
	2	N R_R_ S_L_	E R_R_ S_L_	E R_R_ S_L_
	3	N R_R_ S_L_	E R_R_ S_L_	E R_R_ S_L_
	4	N R_R_ S_L_	E R_R_ S_L_	E R_R_ S_L_
Cadaver 4	1	N R_L_ S_R_	N R_R_ S_L_	N R_L_ S_R_
	2	N R_L_ S_R_	N R_R_ S_L_	N R_L_ S_R_
	3	N R_L_ S_R_	N R_R_ S_L_	F R_L_ S_L_
	4	N R_R_ S_L_	N R_L_ S_R_	N R_L_ S_R_

## Discussion

The diagnoses were correct in all instances except in one cadaver, where a less-experienced resident was unable to appreciate a non-neutral somatic dysfunction at T9/T10. Overall, we found that the palpatory diagnoses were confirmed anatomically in the cadaver as well as radiographically. Furthermore, we found that Fryette’s Principles held to be true in the cadavers when motion in each plane was introduced. Fryette's Principles were developed in 1918 by Harrison Fryette [5]. In brief, the first principle states that with a spine in a neutral position, side bending and rotation will be opposite. In the second principle, in the extended or flexed position, side bending and rotation will be to the same side. Finally, in the third principle, motion in any plane will modify motion in all the other planes. We found that not only were we able to diagnose, but we were able to make adjustments that we believe would have treated the somatic dysfunction based on osteopathic principles in a living patient.

OMT consists of many forms of treatment to improve function by restoring structure and enhancing blood flow. The use of OMT to restore homeostasis has been documented in the literature [6]. The process of successfully performing OMT is foremost dependent upon the practitioner’s appropriate diagnosis in recognizing the somatic dysfunction. The somatic dysfunction affecting the movement of the body part is influenced by muscle, ligament, tendon, joints, and bone and the ease or dis-ease of the anatomical part. The ease or dis-eased part of the structure ultimately affects its function. The cause of the diseased structure can be physiological, e.g. calcium ion (Ca++), creatine phosphokinase (CPK), etc., or anatomical, e.g. lacerations, fractures, burns, etc.

**Figure 3:**
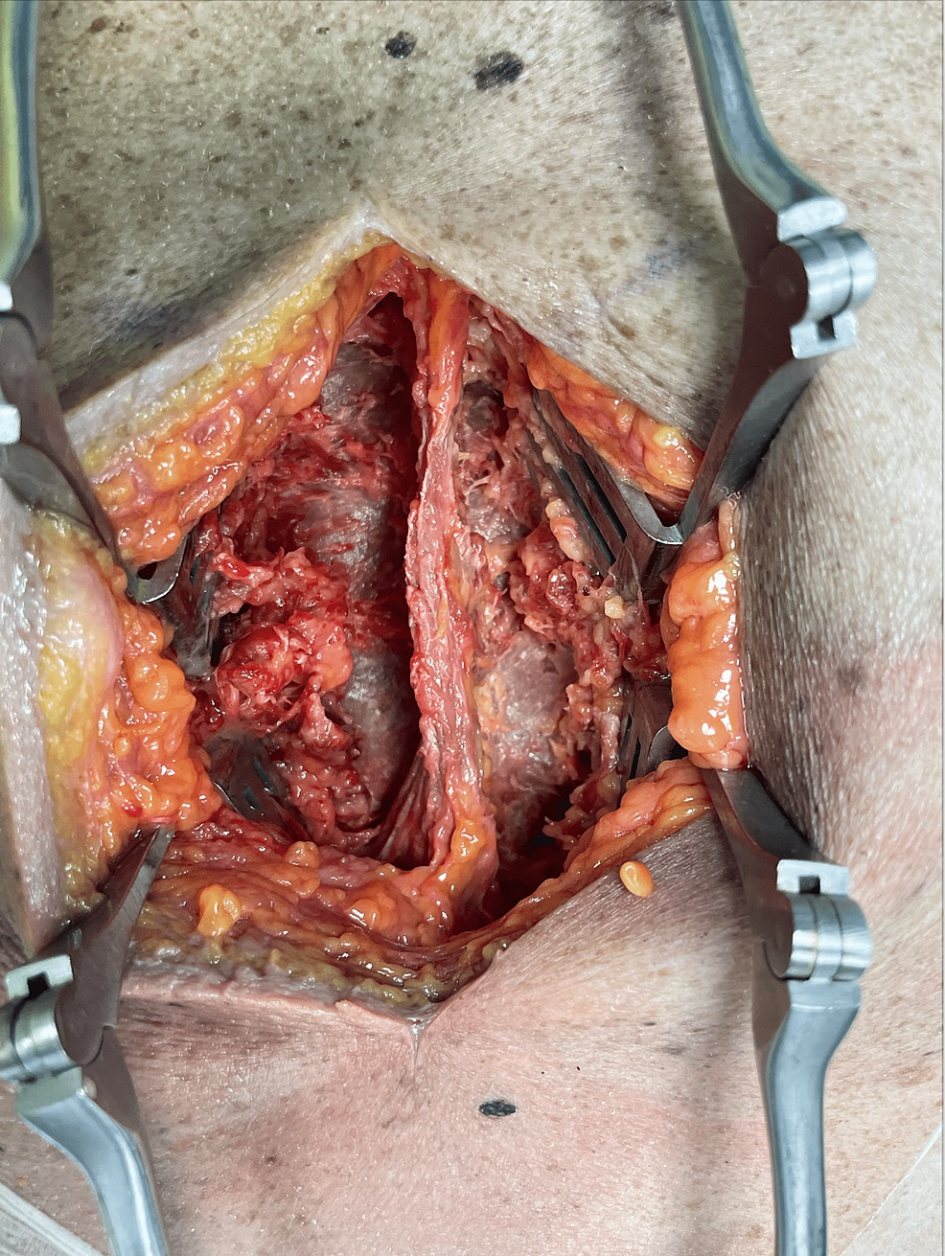
Demonstration of cadaveric thoracic levels T8-T10 with paraspinal musculature removed. Retractors placed after measurements taken under direct vision so as not to move the anatomy.

The function and ease or dis-eased state of the muscle impact the palpation ability of the examiner and often, due to its major contribution to tissue, obscure the structure of other tissues such as skin, ligament, tendon, and bone. It is difficult to completely exclude the state of muscle function and structure when examining the person, even in the most relaxed state, since it is likely that even mere touch can cause a reaction in muscle tissue. Therefore, an experiment was conducted to examine the physician's palpatory skills and the correlation to structure and function when muscle functional state could be excluded following blunt dissection of human cadaver specimen (Figure 3). Fresh refrigerated cadavers were palpated for lesions at the T8, T9, and T10 levels by residents and attendings. Residents who could not consistently diagnose a lesion on a test subject were excluded from providing the results of their findings. This resulted in the PGY1, PGY3, PGY7, and attending results being counted.

After palpation of the cadaver and 100% correlation from the physicians, X-ray fluoroscopy was done to demonstrate anatomical alignment and then the muscle was stripped of the bone sharply without force, movement, and disruption of the bone, ligament, tendons, or joints. It is known that ligamentous structures can be implicated in somatic dysfunction [7]. The physicians reviewed the anatomy and all confirmed the initial lesion diagnosis, which is represented in Table 1. The maintenance of anatomical alignment was then confirmed by repeat X-rays. Next, the anatomy was photographed, and measurements were made of the inter-facet distances listed in Table 2. The lesions all followed Fryette’s Principles as outlined in Table 3 [8]. Next, the lesions were placed in a neutral position from their dis-eased flexion or extension, simulating treatment, and the restoration of anatomical structure returned.

**Table 3:** Fryette's Principles.

Principles	Observation in patients	Types of somatic dysfunction
Principle I	Seen when the spine is in a neutral position. Side bending and rotation occur to opposite sides.	Seen in type I somatic dysfunctions. The entire group curve will be rotated to the side of the convexity.
Principle 2	Seen when the spine is in a non-neutral position (either flexed or extended). Side bending and rotation occur to the opposite side in the restricted segment.	Seen in type 2 somatic dysfunctions. The segment is restricted in motion and becomes worse with either flexion or extension.
Principle 3	When motion is introduced in one plane, it will reduce motion in the other planes.	A dysfunction of motion in one physiologic plane will negatively affect all the other planes of motion of the spine.

The muscles add a great deal of information when palpating for lesions of structure and function. Of course, muscles are not the only contributor to diagnosing an osteopathic lesion. This is apparent when performing the complex osteopathic examination of a patient with a traumatic spinal fracture, dislocation, subluxation, herniation, and traumatic brain injury (TBI) [9]. Although muscle is a large component, it is unknown whether the structure of the underlying anatomy can add much to the physicians’ diagnosis and whether that dis-ease of the structure can be directly restored.

OMT is a multimodal treatment to address the muscle component and restore blood flow to the natural normal state [10]. Though cadaveric studies of osteopathic principles in the thoracic spine have been performed in the past [11], to our knowledge this is the first study to suggest that those physicians who regularly received continuing medical education (CME) for OMT were better able to diagnose spinal somatic dysfunction. The importance of utilizing cadaveric studies cannot be overemphasized. Even Sir William Osler spent his time performing approximately 100 autopsies a year while working at Montreal General Hospital, understanding the importance of this "wet lab" [12]. 

Questions remain, however. Once muscle anatomy and physiology are restored to their natural non-diseased state, can there still be an anatomical component due to the resting physiology of the bone, tendon, ligament complex (BTLC) not due to trauma or tumor? Once the muscle component of the palpatory diagnosis was removed in the cadaver, the visualization, X-ray, and measurements confirmed the dis-eased state of the BTLC. The dis-eased state of the BTLC followed Fryette’s Principles. When the BTLC was returned to a neutral position from flexion or extension, the dis-eased state due to the BTLC was removed.

Overall, we found that the PGY3 resident physicians had the most variability in osteopathic palpatory skills. This is likely because the PGY1 had the least amount of time gap from medical school and formal OMT evaluation. At the other end of the bimodal distribution, PGY7 and attending-level physicians are also adept at evaluating somatic dysfunction, likely owing to years of clinical practice and CME. In our program, residents who would benefit from continued osteopathic manipulative training undergo remediation to enhance their skills. The utility of practicing palpatory skills on cadavers lies in static physiology. In patients, the natural dynamic physiology can cause muscle and tissue texture changes that make demonstrations difficult when patients are assessed by multiple physicians. Therefore, practicing on cadavers allows these same physicians to test their skills in a reliably demonstrative manner. 

These results demonstrate the hallmark osteopathic principle that states direct correlation of structure and function can be restored when the neutral anatomy is restored. This is the anatomic basis for what we do as osteopathic physicians. These results demonstrate that normal physiology is predicated on the function of the BTLC.

## Conclusions

Overall, we have demonstrated that experienced practitioners of OMM and OMT were able to correctly diagnose somatic dysfunction in cadavers most of the time, which was then radiographically and anatomically verified. Again, the reciprocal interrelation between structure and function that is inherent in Fryette’s Principles has been established. The accuracy of diagnoses was diminished in those who have not had as much OMT skill reassessment and CME. We have demonstrated the importance of continuing osteopathic medical education.
